# Lipid Dynamics in Membranes Slowed Down by Transmembrane Proteins

**DOI:** 10.3389/fcell.2020.579388

**Published:** 2020-10-26

**Authors:** Lisa Ebersberger, Torben Schindler, Sonja A. Kirsch, Kristyna Pluhackova, Alexandra Schambony, Tilo Seydel, Rainer A. Böckmann, Tobias Unruh

**Affiliations:** ^1^Physics Department, Institute for Crystallography and Structural Physics, Friedrich-Alexander-Universität Erlangen-Nürnberg, Erlangen, Germany; ^2^Computational Biology, Department Biology, Friedrich-Alexander-Universität Erlangen-Nürnberg, Erlangen, Germany; ^3^Department Biology, Chair of Developmental Biology, Friedrich-Alexander-Universität Erlangen-Nürnberg, Erlangen, Germany; ^4^Science Division, Institut Laue-Langevin, Grenoble, France; ^5^Physics Department, Center for Nanoanalysis and Electron Microscopy (CENEM) and Interdisciplinary Center for Nanostructured Films (IZNF), Friedrich-Alexander-Universität Erlangen-Nürnberg, Erlangen, Germany

**Keywords:** quasielastic neutron scattering, lipid dynamics, protein dynamics, lipid-protein interactions, MD simulations, membrane domains

## Abstract

Lipids and proteins, as essential components of biological cell membranes, exhibit a significant degree of freedom for different kinds of motions including lateral long-range mobility. Due to their interactions, they not only preserve the cellular membrane but also contribute to many important cellular functions as e.g., signal transport or molecular exchange of the cell with its surrounding. Many of these processes take place on a short time (up to some nanoseconds) and length scale (up to some nanometers) which is perfectly accessible by quasielastic neutron scattering (QENS) experiments and molecular dynamics (MD) simulations. In order to probe the influence of a peptide, a transmembrane sequence of the transferrin receptor (TFRC) protein, on the dynamics of 1,2-dimyristoyl-*sn*-glycero-3-phosphocholine (DMPC) large unilamellar vesicles (LUVs) on a nanosecond time scale, high-resolution QENS experiments and complementary MD simulations have been utilized. By using different scattering contrasts in the experiment (chain-deuterated lipids and protonated lipids, respectively), a model could be developed which allows to examine the lipid and peptide dynamics separately. The experimental results revealed a restricted lipid lateral mobility in the presence of the TFRC transmembrane peptides. Also the apparent self-diffusion coefficient of the lateral movement of the peptide molecules could be determined quantitatively for the probed short-time regime. The findings could be confirmed very precisely by MD simulations. Furthermore, the article presents an estimation for the radius of influence of the peptides on the lipid long-range dynamics which could be determined by consistently combining results from experiment and simulation.

## 1. Introduction

The interactions between lipids and proteins in biological membranes play an important role in e.g., cellular signal transport as well as the exchange of molecules or ions between the cell and the cellular environment. A lateral long-range mobility of these membrane components is an essential precondition for many cellular functions, which are initialized by dimerization, formation of lipid-protein-complexes and membrane rafts (Hirsch-Kauffmann et al., 2009). Thereby, membrane dynamics cover a large range of length and time scales. Many biological relevant processes take place on the nanometer length scale (Ramadurai et al., 2009; Busch et al., 2010) and at short times of a few nanoseconds. Consequently, it is not surprising that the complementary methods, quasielastic neutron scattering (QENS) experiments and molecular dynamics (MD) simulations, which cover these short time (from picoseconds up to several nanoseconds) and length (from Ångstrøm up to nanometers) scales, are perfectly suited to observe dynamical processes in membranes (Heller et al., 1993; Niemelä et al., 2010; Jeon et al., 2012; Pluhackova and Böckmann, 2015; Pluhackova et al., 2016b; Lautner et al., 2017; Srinivasan et al., 2019).

Early QENS studies revealed fast localized motions of lipid molecules over short distances (nanometers) and times (a few tens of picoseconds) (Tabony and Perly, 1991; König et al., 1992). These motions differ significantly from the slower long-range diffusion observed by macroscopic methods as e.g., fluorescence recovery after photobleaching (FRAP) (Axelrod et al., 1976; Almeida et al., 1992) or single particle tracking (SPT) (Hsieh et al., 2014). In order to explain these discrepancies, Galla et al. (1979) assigned the concept of the free volume theory (Cohen and Turnbull, 1959; Turnbull and Cohen, 1961, 1970) which originates from glass physics to membranes. They assumed to observe a shaking of lipid molecules in their cage of neighboring lipid molecules in the short-time regime of QENS measurements. The first step of a long-range diffusive motion was thereby explained by the hopping of a lipid molecule out of its cage position between the nearest lipid neighbors into a neighbored vacancy created by thermal heterogeneities (Vaz and Almeida, 1991; Almeida et al., 1992). For many years this so-called free volume theory was able to successfully explain the discrepancies between the diffusion coefficients measured by microscopic and macroscopic methods, respectively. Doubts about the simple mechanistic interpretation of the free volume theory aroused, when realizing by MD simulations and experimental studies that molecular jumps into vacancies could not be observed and neighbored lipid molecules perform highly correlated motions over distances up to the nanometer range (Ayton and Voth, 2004; Gambin et al., 2006; Rheinstädter et al., 2008; Roark and Feller, 2009; Busch, 2012).

In this respect, a new approach to explain the differences between short and long-term dynamics of membrane components was provided by MD simulations. Falck et al. (2008) used atomistic MD simulations of 1,2-dipalmitoyl-*sn*-glycero-3-phosphocholine (DPPC) bilayers in the liquid-crystalline phase and examined the movements of individual lipids in the bilayer, as well as the movements of their neighboring lipid molecules, as a function of time. It was observed, that always when an individual lipid molecule moves very fast into a certain direction its neighboring molecules move essentially into the same direction leading to lipid clusters drifting into the same direction for a certain time. These clusters form, persist for a short time [up to nanoseconds (Falck et al., 2008; Busch et al., 2010)], disintegrate, and randomly rearrange to show up somewhere else again. The resulting movement is referred to as collective flow-like motions of the lipid molecules in a membrane.

Such flow-like lipid motions could also be detected by QENS experiments. Busch et al. (2010); Busch (2012) studied pure multilamellar 1,2-dimyristoyl-*sn*-glycero-3-phosphocholine (DMPC) phospholipid membranes in the liquid-crystalline phase by using various instrumental time resolutions and thus observation times (on the pico to nanosecond time scale). The experiment revealed that the experimentally determined flow velocities match those from the simulation (Busch, 2012). The influence of small molecules as additives and the shape of the phospholipid layer (lamellar, vesicular, monolayers) on the lipid short-time dynamics has also been studied (Busch and Unruh, 2011a; Busch et al., 2012; Pluhackova et al., 2015). At short observation times the collective flow-like motions of the membrane lipids resemble the atomic and molecular movements in liquids (Kegel and van Blaaderen, 2000; Angelini et al., 2011; Cisneros et al., 2011; Zhang et al., 2011; Morhenn et al., 2012, 2013).

Furthermore, Armstrong et al. (2010, 2011) used QENS to study the pico- to nanosecond dynamics of DMPC lipid molecules in D_2_O-hydrated single as well as multilamellar lipid bilayers on silicon wafers. Thereby, a large length scale from 1.3 Åup to 22 Åwas covered, which corresponds to about 3 lipid to lipid distances. Their results indicated a continuous diffusion of the lipid molecules, which changed at distances, shorter than the nearest neighbor distance (<2.37 Å), into short-range flow-like ballistic motions.

While lipid dynamics in membranes is fairly well-understood, the short-time motions of proteins and peptides, respectively, also their influence on lipid dynamics are still unclear despite their relevance for initiating cellular processes. On one hand, various neutron scattering studies are published about antimicrobial peptides as e.g., the bee venom melittin (Sharma et al., 2015, 2016a; Buck et al., 2018) or the so-called Alzheimer peptide amyloid-β (Buchsteiner et al., 2010, 2012; Barrett et al., 2016; Rai et al., 2016) as well as about amyloid fibrils of α-Synuclein (Fujiwara et al., 2019) (Parkinson's disease), which all three tend to damage cell membranes. On the other hand very little is known about the short-time transmembrane dynamics of proteins and peptides in membranes. Niemelä et al. (2010) used MD simulations to study the dynamics of a single Kv1.2 protein embedded in a 1-palmitoyl-2-oleoyl-*sn*-glycero-3-phosphocholine (POPC) lipid bilayer in the liquid-crystalline phase. They observed a kind of transient complex formed by the protein and its neighboring lipids which diffuses laterally in the plane of the membrane. The direct neighboring lipids move together with the protein but are not directly bound to the protein. Diffusion coefficients could be determined for the direct neighboring lipids (*D* ≈ 0.6·10^−8^ cm^2^/s), the other lipids (*D* ≈ 9·10^−8^ cm^2^/s) and the protein itself (*D* ≈ 0.3·10^−8^ cm^2^/s). In the simulation, the protein significantly influenced the dynamics of the surrounding lipids at a distance of about 1–2 nm around the protein. But proteins do not only influence lipid dynamics in their vicinity, they also influence the dynamics of proteins in their neighborhood. MD simulations of a transmembrane receptor CXCR4 have shown that while individual proteins slow down lipids they speed up the dynamics of surrounding proteins prior their dimerization. The fact that this fastening was most significant at low cholesterol concentrations indicate a fascinating complexity in the mutual influence of membrane components on their dynamic properties (Pluhackova et al., 2016b).

In this contribution we present a combined QENS experiment and MD simulation study of the influence of a peptide, more precisely a transmembrane sequence of the transferrin receptor (TFRC) protein, on the dynamics of DMPC lipids in large unilamellar vesicles (LUVs) in the liquid crystalline phase. TFRC plays an important role in the transport of iron into the cell (Moos and Morgan, 2000) as well as in the regulation of the cell iron balance (Gatter et al., 1983; Ponka and Lok, 1999). The receptors, coupled with apotransferrin, accumulate in special membrane areas and can deliver the iron ions to the cell by receptor-mediated endocytosis (Harding et al., 1983). To enable protein accumulation in certain membrane areas, the proteins must be mobile in the lateral plane of the membrane. In order to observe the protein dynamics, vesicles with chain-deuterated DMPC-d54 were studied by QENS in addition to the DMPC lipid vesicles both with and without 6 mol % of the TFRC peptide. This allowed the experimental determination of the lateral self-diffusion coefficient of lipid molecules in absence and presence of the TFRC peptides but also the calculation of the self-diffusion coefficient of the TFRC peptides. Finally, we introduce an estimation of the radius of the peptide influence on the lipid dynamics. Our experimental results of TFRC in DMPC membranes coincide with the findings of Kv1.2 protein embedded in POPC by MD simulations supporting the formation of transient complexes formed by the proteins and its neighboring lipids (Niemelä et al., 2010).

## 2. Materials and Methods

### 2.1. Materials

**Figure 1:**
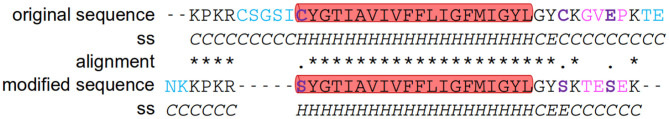
Transferrin transmembrane sequence. Comparison and alignment of the original and the here utilized sequences of the transferrin peptide as well as the prediction of their secondary structure. Sequence alignment was done on the ExPASy webpage using the tool SIM (Miller and Huang, 1991). In order to guide the eye the insertions or deletions are colored blue, substitutions for unsimilar residues are colored magenta and substitutions for similar residues in purpleblue and highlighted in bold. The secondary structure of the peptides was determined using PEP2D (http://crdd.osdd.net/raghava/pep2d/index.html). The part of the peptide predicted to be helical was highlighted by a red background.

**Figure 2:**
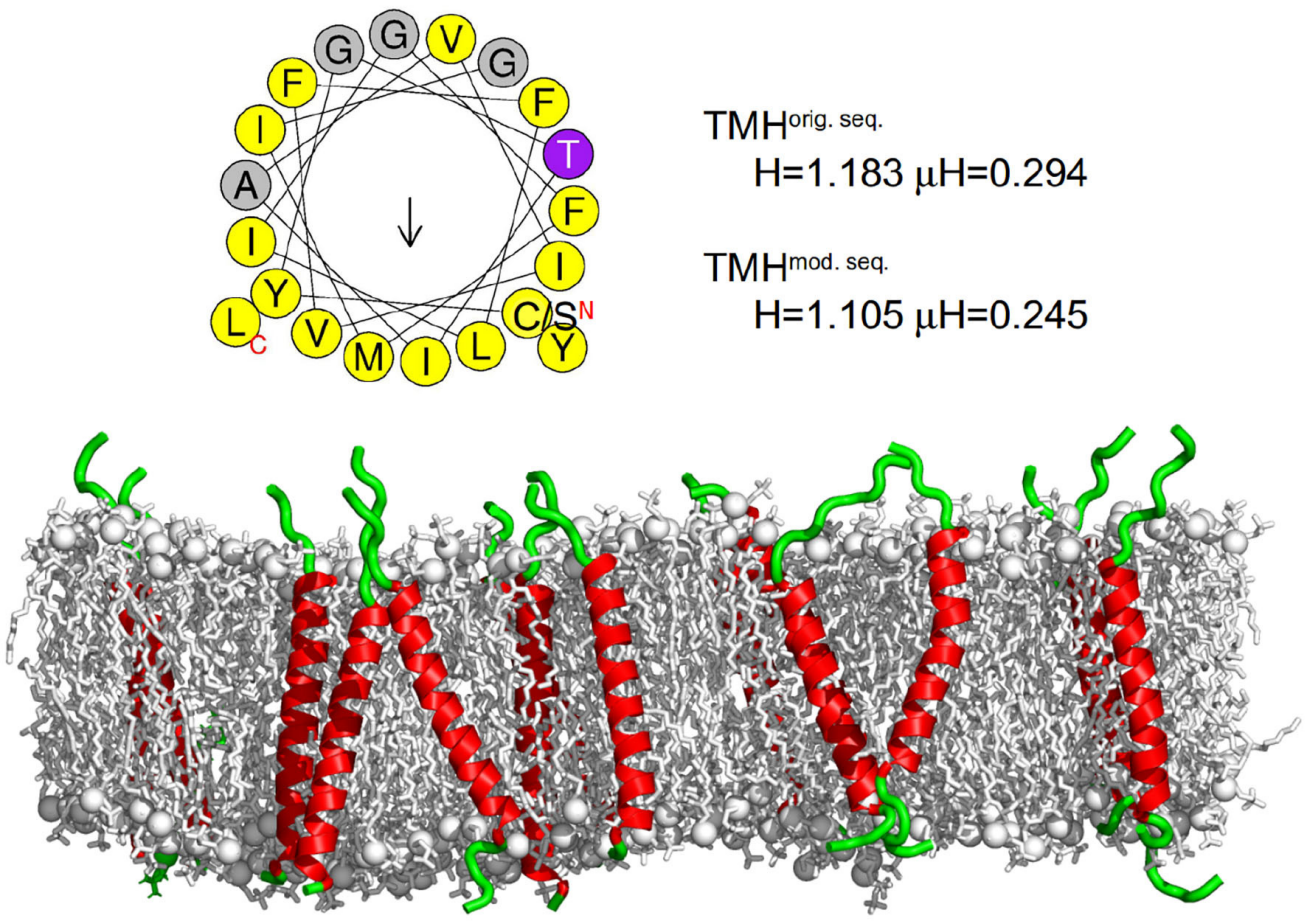
Transferrin transmembrane sequence and structure. **(Top)** Wheel projection of the transmembrane helices (TMH) and the corresponding helicity (H) and hydrophobic moment (μH) for both sequences (please note the only substitution of the N terminal cysteine for serine) as determined by HELIQUEST (Gautier et al., 2008). **(Bottom)** Visualization of the secondary structure of the membrane embedded transferrin TMHs after 500 ns of the MD simulation. The membrane is represented as gray sticks with phosphates highlighted as spheres, the helical part of the peptide is shown in red and the unstructured part in green. The N termini of all peptides are at the bottom and the C termini at the top. On average, transferrin contained 24 helical residues.

Dry DMPC (1,2-dimyristoyl-*sn*-glycero-3-phosphocholine) powder was purchased from Lipoid GmbH (≥98%, Ludwigshafen, Germany). Dry chain-deuterated DMPC-d54 powder was purchased from Avanti Polar Lipids, Inc (>99%, Alabama, United States). The transmembrane sequence of the transferrin receptor (KPKRC SGSIC YGTIAVIVFFLIGFMIGYLGY C KGVEP KTE) turned out to be highly hydrophobic and could not be purified. Thus, the C- and N-termini were slightly modified (NKKPKRS YGTIAVIVFFLIGFMIGYLGY SKTESEK) to increase the solubility and enable purification to ≥85% by PSL GmbH (Heidelberg, Germany). The sequence of the transmembrane helix (TMH) was conserved except for a small replacement at its N terminus of a cysteine for a serine, which did not result in a change of the hydrophobic properties of the transmembrane helix. In Figure 1, both, the original and the modified sequence are displayed in order to elucidate the differences. Thereby, the total hydrophobicity of the peptide reduced from 0.653 to 0.460 [as determined by HELIQUEST (Gautier et al., 2008)] and the charge increased from +3 to +4. Charged residues, which act as membrane anchors and ensure a stable transmembrane orientation, were still present on both ends of the peptide and additionally the transmembrane domain was mostly unchanged. These conditions should ensure, that the transmembrane orientation is supposed to be preserved for the new sequence. Potentially, the aggregation behavior of the peptides could be influenced by the deletion of the patch of the polar residues (5-CSGSI-9), however, a peptide aggregation was not observed on the simulation time scale (Figure 2 bottom). This peptide was used for the experiments reported here. Additionally, for vesicle preparation, chloroform (CHCl_3_, ≥99%) from Carl Roth GmbH (Karlsruhe, Germany) and deuterated water (D_2_O, ≥99.9%) from Euriso-Top (Saarbrücken, Germany) was utilized.

### 2.2. Sample Preparation

Two samples containing pure lipid vesicles (DMPC and DMPC-d54, respectively) and two analogous samples with additional 6 mol % (with respect to the lipid concentration) of the TFRC peptide sequence were prepared.

For the lipid vesicle preparation, 20 mg chloroform (CHCl_3_) were added to 1 mg dry phospholipid powder. The solution was vortexed for at least 3 min until a macroscopically transparent liquid phase formed. The solvent was removed by using a rotary evaporator yielding a lipid film at the surface of the round bottom flask. To remove remaining traces of the solvent, the flask was evacuated for at least 12 h. Subsequently, the dry lipid film was hydrated with 90 wt % D_2_O relative to the lipid weight at *T* = 40°C and vortexed for 3 min. The lipid-water-mixture was stored in a hot cabinet at 40°C for 2 h to enable swelling of the lipid. Finally, three freeze-thaw cycles were performed by alternately storing the sample for 10 min at −20°C in a fridge and for 15 min at 50°C in the hot cabinet.

LUVs were produced by 21 repeated extrusion cycles using an extruder from Avestin Europe GmbH (Mannheim, Germany) and 1 ml syringes from Hamilton Germany GmbH (Gräfelfing, Germany). During the extrusion process, the extruder was placed on a heating plate (at 50°C) to ensure that the temperature of the lipid-water-mixture remains above the gel/fluid phase transition temperature. For the extrusion a filter with a pore diameter of 100 nm was used yielding LUVs with diameters of about 120 nm (determined by photon correlation spectroscopy, PCS). For preparing lipid-peptide vesicles, the 6 mol % of peptide relative to the lipid concentration was added as a powder to the lipid vesicle solution. The amount of 6 mol % of TFRC was chosen to guarantee a sufficient scattering contribution of the peptides and therefore enable to study not only the influence of peptides on the lipid dynamics but also the peptide dynamics itself.

### 2.3. Quasielastic Neutron Scattering

QENS experiments (Lautner et al., 2015) were performed at the backscattering spectrometer IN16B (Frick et al., 2010; Henning et al., 2011) at Institut Laue-Langevin (ILL) in Grenoble, France. For the experiments, an instrumental resolution (full width at half maximum) of ΔE = 0.75 μeV, an elastic wavelength of 6.271 Å [Si(111)], and a sinus Doppler velocity profile with a maximum speed of 4.55 m/s was chosen. During the measurement, the sample temperature was controlled and set to 310 K using a cryofurnace. Approximately 1 ml of sample was needed to fill the hollow cylindrical aluminum container with a gap distance of 0.2 mm. The corresponding effective sample thickness of about 0.63 mm has been demonstrated to result in experimental data with negligible multiple scattering effects (Busch and Unruh, 2011b). Each sample as well as pure D_2_O and the empty can was measured for 5 h. For determination of instrument resolution and detector sensitivity, a vanadium foil with a thickness of 0.2 mm placed inside another aluminum hollow cylinder sample holder was measured for 5 h.

The raw data were treated using the program LAMP (LAMP, 2018) for summing up consecutive measurement files, normalizing to primary beam intensity, and removing defect detector signals. For sample, vanadium, and D_2_O measurements, absorption correction and subtraction of the empty can measurement were performed, respectively. The intensity of the vanadium elastic line was integrated, corrected for the Debye-Waller factor, and used for normalization of the detector sensitivities. The corrected spectra were converted to scattering functions *S*(*Q*,ω) while binning the energy transfer data to equidistant steps of 0.1 μeV. Finally, the D_2_O spectrum scaled according to the amount of D_2_O in the sample was subtracted from the sample data.

For data analysis the program FABADA (Pardo et al., 2011, 2014) has been used which is based on a Bayesian analysis. This program performs a convolution of a theoretical model function with the experimental instrument resolution function when fitting the parameters of the model function to the experimental data. The algorithm performed 1,000,000 cycles with an automatic adjustment of the step size every 100,000 cycles with a convergence factor of 0.66. The advantage of the Monte Carlo based approach is that the probability distribution function (PDF) of the figure of merit (here χ^2^) is determined for each optimization allowing to identify the quality of two models with respect to each other.

The theoretical model function describes the molecular dynamics in pure lipid vesicles by localized slow and fast intra-molecular motions and a long-range mobility (Bée, 1988; Busch et al., 2010; Busch, 2012). The long-range mobility can be either described by a Lorentzian *L*(Γ_lipid_,ω) characterizing a diffusive lipid motion or by a Gaussian *G*(σ_lipid_,ω) describing a flow-like motion. The fast localized motion has been found to be too fast to be distinguished from a constant contribution *c* to *S*(*Q*,ω) within the narrow dynamic range of the measurement whereas the slow localized motion is characterized by the Lorentzian half width at half maximum (HWHM) Γ_s_. For diffusive long-range motions, the scattering function *S*_diff_(*Q*,ω) can be modeled as

(1) $$\begin{array}{l} S_{\text{diff}}(Q,\omega )=a(Q)\cdot L(\Gamma_{\text{lipid}},\omega )⊗[A_{\text{s}}(Q)\cdot \delta (\omega )\\ \;+\;\left(1-A_{\text{s}}(Q))\cdot L(\Gamma_{\text{s}},\omega \right)]+c\;, \end{array}$$

with delta functions δ(ω), the Lorentzians

(2) $$\begin{array}{l} L\left(\Gamma_{\text{s, lipid}},\omega \right)=\frac{1}{\pi }\cdot \frac{\Gamma_{\text{s, lipid}}}{\Gamma_{\text{s, lipid}}^{2}+\omega^{2}}\;, \end{array}$$

the elastic incoherent structure factor (EISF) *A*_s_(*Q*) of the respective slow localized motion, and a prefactor *a*(*Q*) containing the Debye-Waller factor. The EISF provides insight into the amplitude and geometry of the localized motion (Busch et al., 2010). From the HWHM $\Gamma_{\text{lipid}}\left(Q\right)=\hbar DQ^{2}$ the apparent self-diffusion coefficient *D* of the lipids can be calculated. It characterizes the lateral motion of the lipid molecules within the membrane within the observation time given by the resolution of the spectrometer.

Accordingly, for a flow-like motion, the scattering function *S*_flow_(*Q*,ω) can be modeled as

(3) $$\begin{array}{l} S_{\text{flow}}(Q,\omega )=a(Q)\cdot G(\sigma_{\text{lipid}},\omega )⊗[A_{\text{s}}(Q)\cdot \delta (\omega )\\ \;+\left(1-A_{\text{s}}(Q))\cdot L(\Gamma_{\text{s}},\omega \right)]+c\;, \end{array}$$

with the Gaussian

(4) $$\begin{array}{l} G\left(\sigma_{\text{lipid}},\omega \right)=\frac{1}{\sigma_{\text{lipid}}\sqrt{2\pi }}\exp\left[-\frac{\omega^{2}}{2\sigma_{\text{lipid}}^{2}}\right]\;, \end{array}$$

the standard deviation σ_lipid_ = ℏ*v*_0_*Q*, and the most probable flow velocity *v*_0_.

For vesicles with incorporated peptides, the peptide dynamics is modeled by a fast localized motion, reflecting CH3 group rotations and other fast internal movements and a long-range diffusive motion [Lorentzian *L*(Γ_peptide_,ω)]. It is assumed that the peptide spans both membrane leaflets which should lead to slower long-range self-diffusion compared to the lipid molecules as well as disturbing and not participating in the collective lipid molecular flows within the single lipid monolayers.

Combining the additive scattering functions of the lipid (assuming flow-like long-range motion) and peptide diffusion leads to

(5) $$\begin{array}{l} S_{\text{flow}}(Q,\omega )=p\;\cdot \;b(Q)\cdot L(\Gamma_{\text{peptide}},\omega )⊗[B_{\text{s}}(Q)\cdot \delta (\omega )\;\\ \;+\left(1-B_{\text{s}}(Q))\cdot L(\Gamma_{\text{s,peptide}},\omega \right)]+\\ \;(1-p)\cdot a(Q)\cdot G(\sigma_{\text{lipid}},\omega )⊗[A_{\text{s}}(Q)\cdot \delta (\omega )\\ \;+\left(1-A_{\text{s}}(Q))\cdot L(\Gamma_{\text{s,lipid}},\omega \right)]+c. \end{array}$$

The leading coefficients *p* and (1 − *p*) represent the scattering contributions of the peptides and lipids, respectively. *B*_s_(*Q*) represents the EISF of the peptides and *b*(*Q*) denotes a coefficient for the local peptide motion according to *a*(*Q*) for the lipids. All localized internal motions that are too fast to be distinguished from a scattering contribution constant in *Q* are summed in constant *c*.

For long-range lipid diffusion one gets accordingly.

(6) $$\begin{array}{l} S_{\text{diff}}(Q,\omega )=p\;\cdot \;b(Q)\cdot L(\Gamma_{\text{peptide}},\omega )⊗[B_{\text{s}}(Q)\cdot \delta (\omega )\\ \;+\left(1-B_{\text{s}}(Q))\cdot L(\Gamma_{\text{s,peptide}},\omega \right)]+\\ (1-p)\cdot a(Q)\cdot L(\Gamma_{\text{lipid}},\omega )⊗[A_{\text{s}}(Q)\cdot \delta (\omega )\\ \;+\left(1-A_{\text{s}}(Q))\cdot L(\Gamma_{\text{s,lipid}},\omega \right)]+c. \end{array}$$

### 2.4. Molecular Dynamics Simulations

Molecular dynamics (MD) simulations of a DMPC phospholipid bilayer with and without 6 mol% of the TFRC transmembrane sequence, respectively, were performed using the software package GROMACS (van der Spoel et al., 2005; Pronk et al., 2013) and the CHARMM36 force field (Klauda et al., 2010; Best et al., 2012). The pure DMPC membrane was simulated for 300 ns and mixed DMPC/TRFC systems for 500 ns. The simulated box sizes amount to 15 × 15 × 9 nm (DMPC bilayers) and 15 × 15 × 8 nm (DMPC/TFRC bilayer), respectively, containing either 790 DMPC molecules (pure DMPC system) or 576 (768) DMPC molecules plus 36 (16) TFRC transmembrane peptides which corresponds to a ratio of 16 and 48 lipids per peptide molecule, respectively. The systems were equilibrated at 310 K meeting the temperatures of the corresponding QENS experiments. A detailed description of the parameters and simulation setups can be found in Pluhackova et al. (2016a) and Sandoval-Perez et al. (2017), respectively.

For the determination of the lipid and peptide self-diffusion coefficients, the mean square displacement (MSD) of the lipid and peptide atoms, respectively, was calculated as a function of time. To compute the MSD, the simulations were splitted into time intervals of 60ns. The linear range of the MSD vs. time curve was selected (2–5 ns) and the diffusion coefficient was determined from the slope of the corresponding linear regression line (Böckmann et al., 2003).

## 3. Results

### 3.1. Lipid and Peptide Dynamics Within DMPC and DMPC-d54 LUVs

In order to study the lipid and peptide dynamics in bilayers, LUVs consisting of DMPC and chain-deuterated DMPC-d54, respectively, were measured in the fluid phase, each with and without 6 mol% of TFRC. The deuterated DMPC-d54 was chosen to reduce the scattering signal of the lipids to study the peptide dynamics. Since the peptide diffusion was expected to be rather slow, the QENS experiments were performed at the backscattering spectrometer IN16B which provides a high energy resolution (Δ*E*_FWHM_ ≈ 0.75 μeV) corresponding to an observation time *t*_obs_ of about 5 ns.

**Figure 3:**
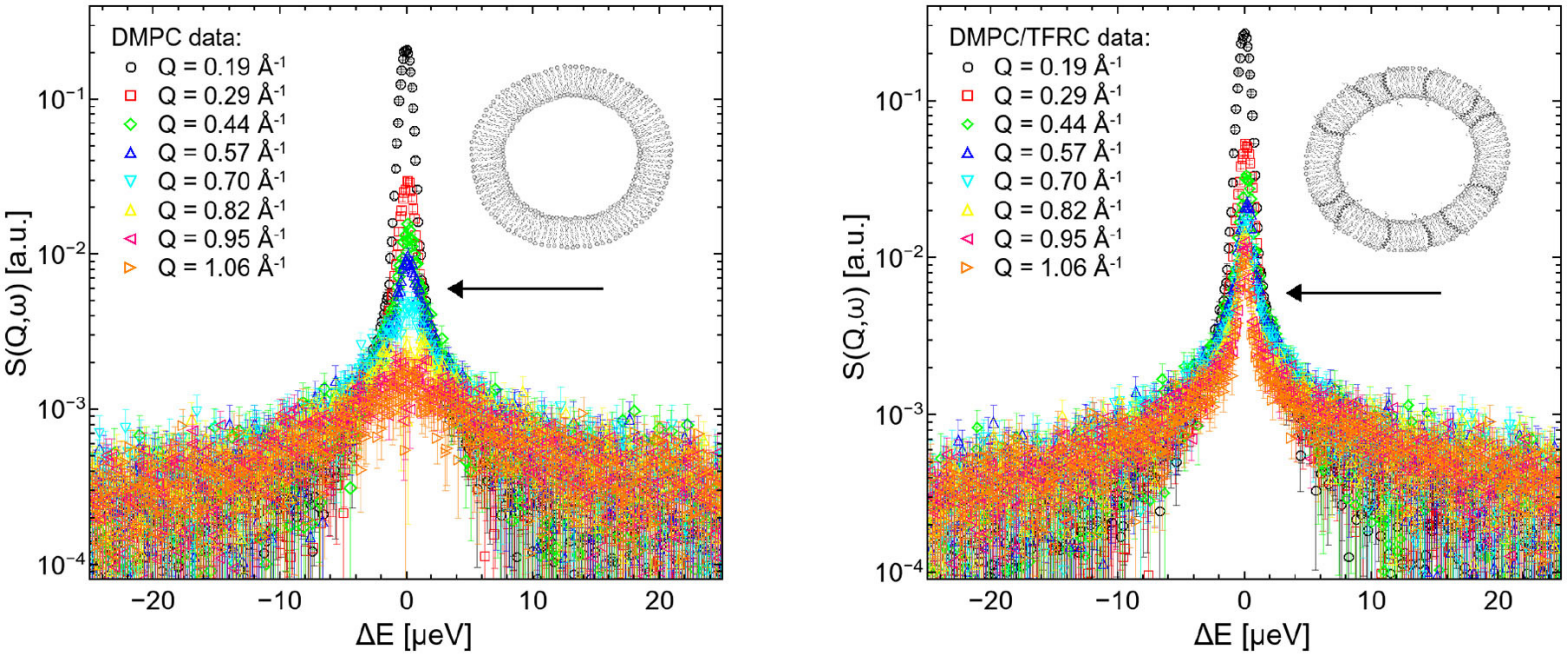
QENS spectra of DMPC **(left)** and DMPC/TFRC **(right)** LUVs plotted for different *Q*-values.

QENS spectra of pure DMPC and DMPC/TFRC LUVs are visualized in Figure 3. Comparing the broadening of the elastic line for corresponding Q-values with the pure DMPC LUVs, it can be observed that the peptide loaded LUVs (right panel) exhibit a significantly reduced line width. This clearly indicates a slowdown of the overall molecular dynamics in the peptide loaded vesicles. The question arises whether this slowdown can be attributed solely to the slow peptide dynamics or whether the presence of the peptides also induces a reduction of the lateral dynamics of the lipid molecules.

To answer this question, it is necessary to test if the lipid long-range motions exhibit either a diffusive or a flow-like behavior on the studied time-scale. For this purpose the QENS data for pure DMPC and deuterated DMPC-d54 lipids haven been analyzed.

**Figure 4:**
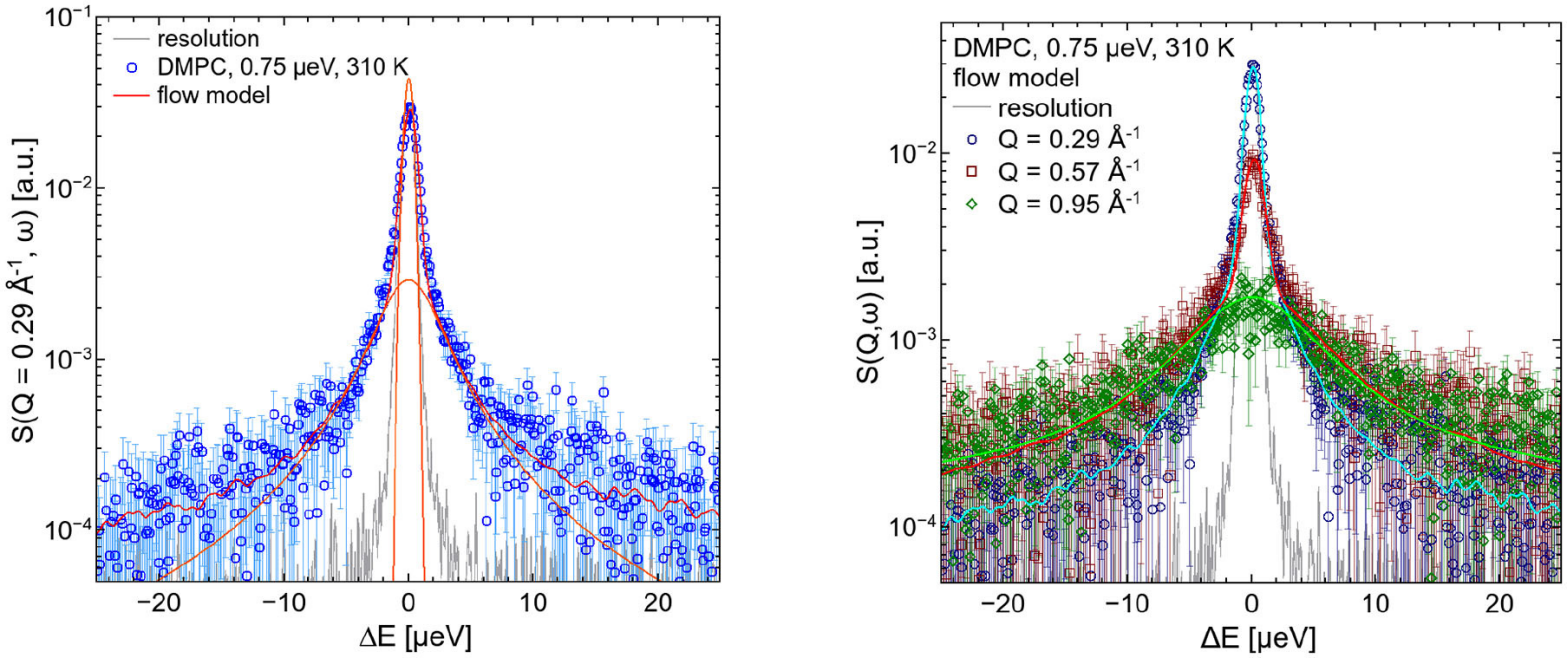
QENS spectra of DMPC LUVs. The experimental data for *Q* = 0.29 Å^−1^ are plotted in the left panel as blue diamonds. The best fit of the flow model (Equation 3, red) and the corresponding additive components of the fit are visualized (orange). In the right panel, three spectra and corresponding best fits are displayed for *Q* = 0.29 Å^−1^, *Q* = 0.57 Å^−1^, and *Q* = 0.95 Å^−1^. The instrumental resolution is plotted in gray.

**Figure 5:**
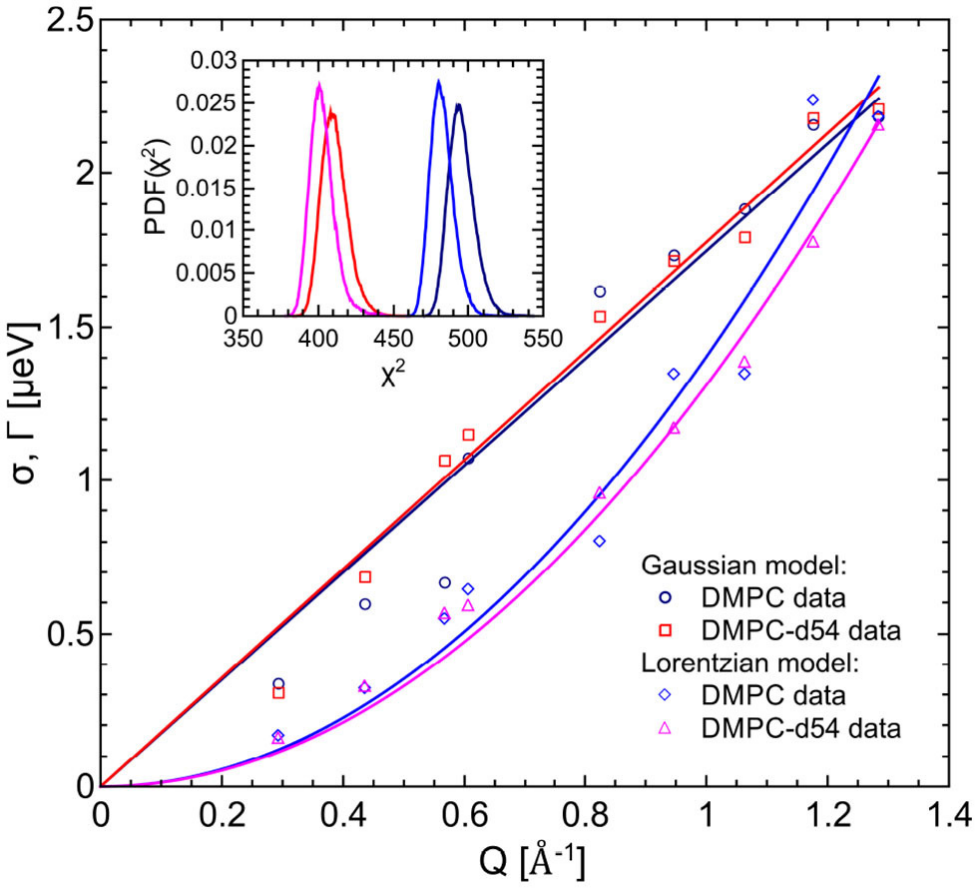
PDFs as a function of χ^2^ for the fit model of the respective DMPC (blue) and DMPC-d54 (red) data. The diffusion (Lorentzian) model better represents the long-range lipid dynamics which can be seen by the PDF maxima at smaller χ^2^-values. Furthermore, the standard deviations and HWHM are plotted as functions of *Q*.

The left hand plot of Figure 4 represents the QENS spectra of the measurement of pure DMPC LUVs fitted with the flow model (cf. 3) for *Q* = 0.29 Å^−1^. The best fit to the data is plotted (red line) including the individual additive components of Equation (3). Three additional spectra for *Q* = 0.29 Å^−1^, *Q* = 0.57 Å^−1^, and *Q* = 0.95 Å^−1^ are displayed in the right hand panel of Figure 4 with the corresponding best fits. The model of flow-like motions correctly describes the data over the whole observed *Q*-range. The same applies for the model of diffusive movements. The long-range lipid dynamics in DMPC LUVs can therefore be modeled by both, flow-like and diffusive motions. For a better comparison of the fit results, the respective PDFs of the two models are displayed in the Figure 5 with the corresponding standard deviations and half-widths of the DMPC and DMPC-d54 spectra. From the PDFs it is obvious that the model of diffusive motions fits the DMPC and the DMPC-d54 data better than the flow model. For both sample systems, the maximum and the center of mass of the PDF is found for the diffusive model at smaller χ^2^. From this result, it is concluded that for an observation time as short as *t*_obs_ ≈ 5 ns the lipid long-range motion is described best by a diffusion process.

The results of the calculated most probable velocities as well as the apparent self-diffusion coefficients obtained from the fits to the data described above are listed in Table 1. From the data it can be extracted that the deuteration of the lipid chains has no influence on the long-range dynamics of the lipids molecules. This applies to both tested models, the diffusion and the flow model (Equations 1 and 3). The calculated diffusion coefficients for lipid molecules in both, pure DMPC and DMPC-d54 vesicles, amount to about 20·10^−8^ cm^2^/s. Diffusion coefficients of nearly comparable systems are found to be more than two times the value presented here. In a system of multilamellar DMPC bilayers at 303 K, measured at an instrumental resolution of about 4 μeV, the calculated diffusion coefficients for the lipid molecules was found to be 44·10^−8^ cm^2^/s (Busch et al., 2010). Another system of DMPC lipid vesicles at 310 K (instrumental resolution of about 3.4 μeV) indicated a diffusion coefficient of about 50·10^−8^ cm^2^/s (Sharma et al., 2016b). It has to be clarified, that diffusion coefficients determined by measurements at different neutron scattering instruments, and thus different instrumental resolutions, are not directly comparable. But it is known, that with decreasing instrumental resolution, and therefore increasing observation times, the diffusion coefficients tend to slow down (Barrett et al., 2016; Lautner et al., 2017). However, the reported DMPC diffusion coefficient of 20·10^−8^ cm^2^/s in this article is still more than two times the long-range diffusion coefficient of macroscopic measurements [e.g., FRAP 8.4·10^−8^ cm^2^/s (Cevc, 1993)].

**Table 1:** Results of the neutron backscattering measurements of DMPC and chain-deuterated DMPC-d54 vesicles, with and without the respective amount of 6 mol% of TFRC.

	**DMPC**	**DMPC-d54**	**DMPC/TFRC**	**DMPC-d54/TFRC**
Lipid *v*_0_ [m/s]	0.27	0.27	0.13	0.13
TFRC *D* [cm^2^/s]	–	–	0.4·10^−8^	0.5·10^−8^
Lipid *D* [cm^2^/s]	21·10^−8^	20·10^−8^	8·10^−8^	8·10^−8^
TFRC *D* [cm^2^/s]	–	–	0.5·10^−8^	0.5·10^−8^

*The resulting parameters for using Equation (5) (flow model for the lipids, upper two rows) and Equation (6) (diffusion model for the lipids, lower two rows). The long range diffusion of the peptide sequence is hardly affected by neither the evaluation model nor the deuteration of the lipids*.

**Figure 6:**
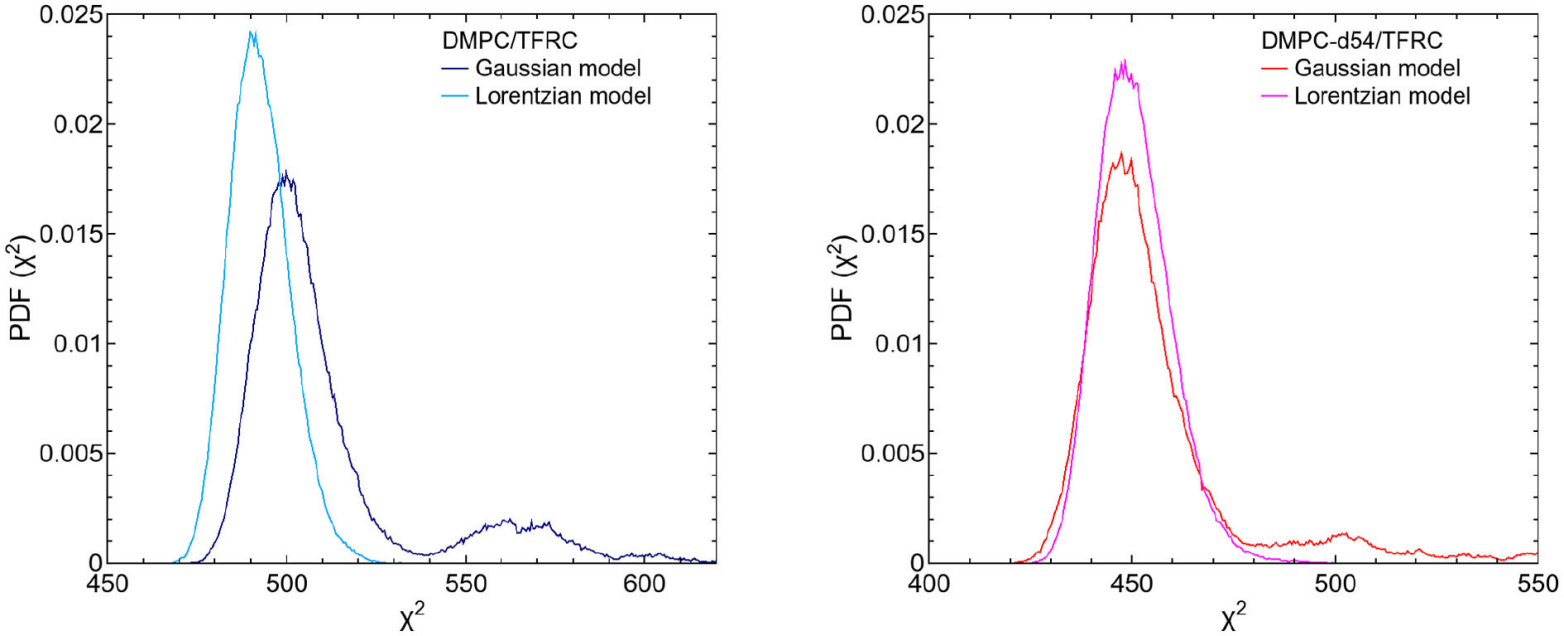
PDFs as a function of χ^2^ for both fit models (Gaussian and Lorentzian model) of the respective DMPC/TFRC (light blue/dark blue) (left figure) and DMPC-d54/TFRC (magenta/red) (right figure) data. Regarding the DMPC/TFRC data, the diffusion (Lorentzian) model, again, slightly better represents the long-range lipid dynamics compared to the flow (Gaussian) model.

**Figure 7:**
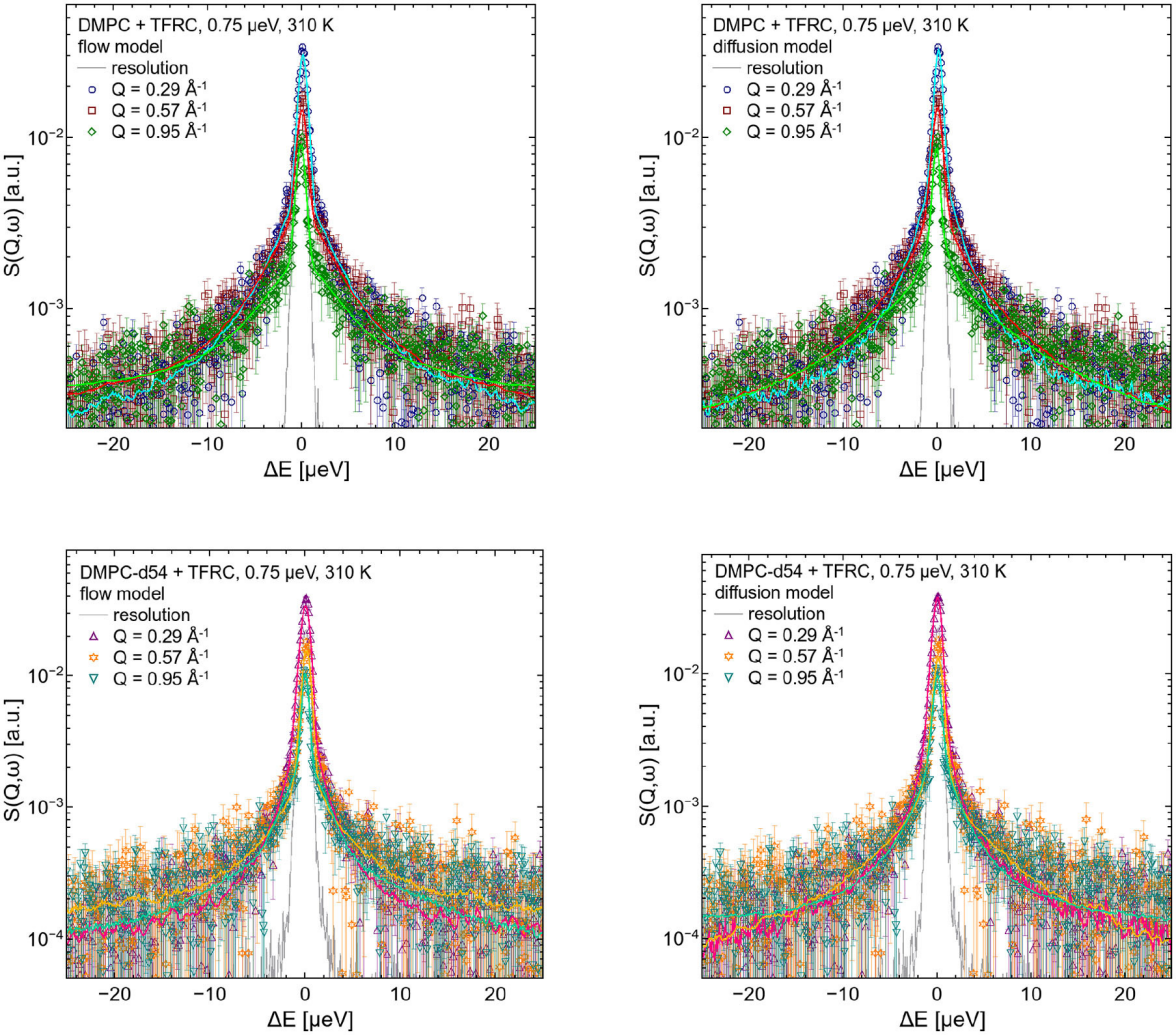
QENS spectra of DMPC/TFRC LUV dispersions for *Q* = 0.29 Å^−1^, *Q* = 0.57 Å^−1^, and *Q* = 0.95 Å^−1^. In the left hand figures, the DMPC/TFRC LUV data (colored symbols) are displayed including the best fits of the flow model (colored lines, top: DMPC, bottom: DMPC-d54). In the right hand figure the same data and corresponding best fits of the diffusion model are visualized. The instrumental resolution is plotted as gray line in all panels.

**Figure 8:**
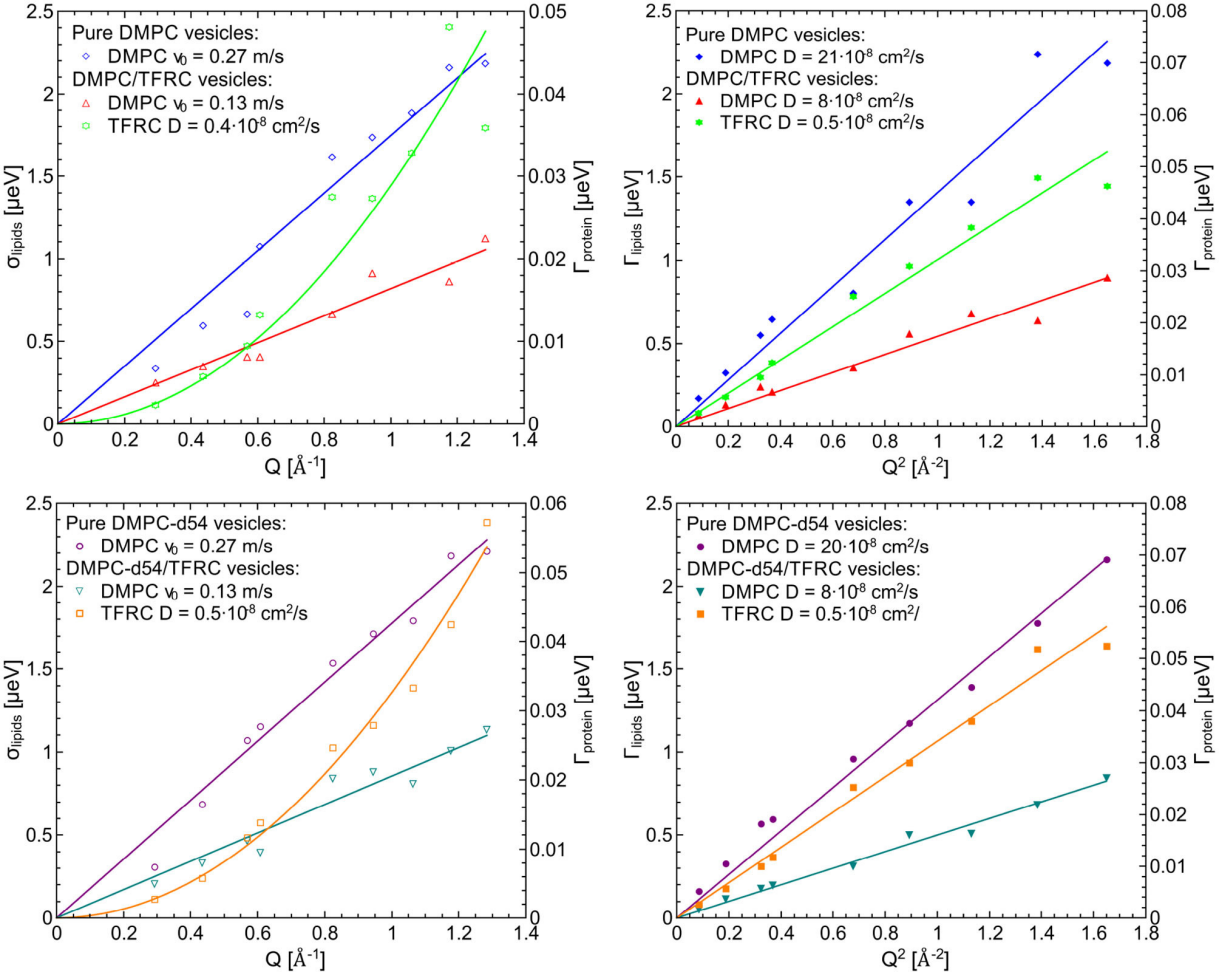
Left figures (lipid flow model): σ-values for the lipid flow motion resulting from QENS data of pure DMPC LUVs and DMPC/TFRC LUVs (top) as well as DMPC-d54 LUVs and DMPC-d54/TFRC LUVs (bottom) and Γ-values of the diffusive motion of the TFRC peptide sequence (green and orange curves, right scale) as a function of *Q*, right figures (lipid diffusion model): Γ-values for the lipid and the peptide, respectively, of the same samples as a function of *Q*^2^.

For the evaluation of the QENS data of the peptide loaded LUVs the corresponding flow (Equation 5) and diffusion (subsection 2.3) models for the lipid motion have been used. For DMPC LUVs (6 mol% TFRC), the scattering contribution of the peptide with respect to the total scattering of the sample is about 20%. For the chain-deuterated DMPC-d54 LUVs, this scattering contribution amounts to almost 50%. The respective PDFs as functions of χ^2^ are displayed in Figure 6 for each model (Gaussian and Lorentzian) and for both, the DMPC/TFRC data as well as the DMPC-d54/TFRC data. A careful analysis of both data sets allowed the extraction of the peptide dynamics. The DMPC/TFRC spectra are plotted for different *Q*-values in Figure 7 with fitting curves for both analytical models. The σ-values of the lipid long-range flow motion resulting from the analysis of the QENS data of pure DMPC LUVs and DMPC/TFRC LUVs with the flow model are plotted as a function of *Q* in the left hand panel of Figure 8. In the right hand part of this figure the Γ-values of the lipid long-range diffusive motion acquired from the analysis with the diffusion model for the same samples are plotted as a function of *Q*^2^. From the slopes of the respective linear regression to the data, the most probable velocities *v*_0_ and the self-diffusion coefficients *D* of the lipids in the presence of peptides and the *D*-values for the peptides have been determined and are summarized in Table 1.

The results clearly indicate that the long-range lipid dynamics is more than halved in presence of TFRC peptides compared to the lipid mobility in pure lipid vesicles. The most probable velocity of lateral lipid motions slows down from 0.27 to 0.13 m/s in the presence of the peptides and the observed apparent self-diffusion coefficient decreases from 21·10^−8^ to 8·10^−8^ cm^2^/s. Although it has been demonstrated above that the lipid diffusion model represents the data slightly better than the flow model, both pictures essentially characterize the long-range lipid motion consistently well. A reduced lipid mobility in the presence of the TFRC peptide results for both models. It is assumed that the transmembrane peptides are hindered in their diffusion not only by their larger mass compared to the lipids but also by the anchoring in both membrane leaflets. Thus, a certain amount of lipid molecules neighbored to the peptides is likely to also be hindered in their long-range mobility which leads to the decrease of their self-diffusion coefficient.

It is remarkable that no difference in the results for the DMPC and chain-interpreted DMPC-d54 dynamics could be detected (cf. Table 1). It is also interesting that the relatively small scattering contribution of the peptides of about 20% in the non-deuterated DMPC vesicles is already sufficient to analyze the peptide dynamics satisfactorily. The self-diffusion coefficient of the peptides could be determined and amounts to $D_{\text{peptide}}=0.5\cdot 10^{-8}$ cm^2^/s. This value is about 40 times smaller than the self-diffusion coefficients of the lipids and supports the interpretation of the observed reduced lipid long-range mobility in presence of TFRC. The observed small value of the self-diffusion coefficient of the peptide is not only independent of the model used to describe the lipid long-range motion but also of the contrast variation by deuteration of the lipid chains. This illustrates very nicely the excellent reproducibility and reliability of the QENS measurements.

These results demonstrate for the first time, that QENS combined with an observation time of *t*_0_ ≈ 5 ns is capable to determine the self-diffusion coefficient of TFRC in a DMPC membrane. In comparison, the diffusion of TFRC peptides has been studied before by other methods like FRAP and SPT. Srivastava and Petersen (1998) studied the lateral diffusive motion of labeled TFRC monomers in 3T3 fibroblasts as well as in HEp2 carcinoma cells by using FRAP experiments at 298 K. The resulting TFRC diffusion coefficients in 3T3 amounts to 0.68±2.1·10^−8^ cm^2^/s and in HEp2 0.29±1.3·10^−8^ cm^2^/s, respectively. Thereby, a free Brownian motion of the proteins can be observed in the lateral plane of the membrane as long as their mobility is not hindered by interactions with slower membrane components or immobile molecules. The diffusion coefficient of TFRC has also been determined by SPT measurements in a plasma membrane (Sako and Kusumi, 1994, 1995). A value of ~10^−9^ cm^2^/s has been reported.

In conclusion, it can be stated that the values for the self-diffusion coefficient of the TFRC peptide observed by QENS on short time scales are in the same range as those observed by FRAP and SPT under similar conditions on extended time scales. Obviously, the long-range mobility of the peptide reaches its long-time diffusion limit already within an observation time as short as 5 ns.

### 3.2. Complementing the QENS Results by MD Simulations

A more detailed analysis of the QENS data can be achieved using the results of MD simulations which in turn can be validated by a comparison with QENS data. Thus, state-of-the-art MD simulations have been performed for DMPC lipid bilayers with 2, 6 mol%, and without TFRC. The self-diffusion coefficients of the DMPC lipids and the peptides have been determined. The corresponding results are listed in Table 2. The diffusion coefficients of the lipids are in excellent agreement with the respective QENS results. This holds for pure DMPC membranes ($D_{\text{QENS, DMPC}}=21\cdot 10^{-8}$ cm^2^/s) and for DMPC membranes loaded with 6 mol% TFRC ($D_{\text{QENS, DMPC, TFRC}}=8\cdot 10^{-8}$ cm^2^/s).

**Table 2:** Lateral molecular self-diffusion coefficients of DMPC and TFRC derived from MD simulations of DMPC lipid membranes with 2, 6 mol%, and without TFRC, respectively.

	**Pure DMPC**	**DMPC with 2 mol% TFRC**		**DMPC with 6mol% TFRC**	
		**DMPC**	**TFRC**	**DMPC**	**TFRC**
*D* [cm^2^/s]	18.95·10^−8^	13.30·10^−8^	6.02·10^−8^	7.89·10^−8^	3.98·10^−8^
	±0.09·10^−8^	±0.06·10^−8^	±0.15·10^−8^	±0.04·10^−8^	±0.07·10^−8^

However, in the MD simulation the transmembrane peptides exhibit a significantly faster lateral diffusion than observed experimentally ($D_{\text{QENS, TFRC}}=0.5\cdot 10^{-8}$ cm^2^/s). The low peptide mobility observed experimentally in the peptide-dense system (6 mol% TFRC) suggests peptide-lipid domain formation as a possible reason for this discrepancy. A corresponding formation of TFRC clusters has been reported before by Srivastava and Petersen (1998). The formation of domains with large peptide concentration is unlikely to be observed in atomistic MD simulations on the submillisecond timescale. On the basis of the QENS data, such a domain formation can, however, not easily be distinguished from the more local demixing of the lipids around each peptide in the membrane as assumed by our QENS data fitting model.

### 3.3. Lipid-Peptide Interaction Length

The self-diffusion coefficients determined by QENS characterize the average mobility of the corresponding molecular species. To which degree is the dynamics of lipid molecules influenced by the peptide? Up to what distance does the peptide influence the lipid dynamics? In order to estimate this averaged interaction radius of the peptide influence on the lipid dynamics, the flow model represented by Equation (5) has been extended by replacing the scattering function of the lipid motions by two additional terms:

(7) $$\begin{array}{l} S_{\text{flow}}(Q,\omega )=p\;\cdot \;b(Q)\cdot L(\Gamma_{\text{peptide}},\omega )⊗[B_{\text{s}}(Q)\cdot \delta (\omega )\\ \;+\left(1-B_{\text{s}}(Q))\cdot L(\Gamma_{\text{s,peptide}},\omega \right)]+\\ (1-p)\cdot l\cdot a(Q)\cdot G(\sigma_{\text{lipid,1}},\omega )⊗[A_{\text{s}}(Q)\cdot \delta (\omega )\\ \;+\left(1-A_{\text{s}}(Q))\cdot L(\Gamma_{\text{s,lipid}},\omega \right)]+\\ (1-p)\cdot (1-l)\cdot a(Q)\cdot G(\sigma_{\text{lipid,2}},\omega )⊗[A_{\text{s}}(Q)\cdot \delta (\omega )\\ \;+\left(1-A_{\text{s}}(Q))\cdot L(\Gamma_{\text{s,lipid}},\omega \right)]+c\;. \end{array}$$

**Figure 9:**
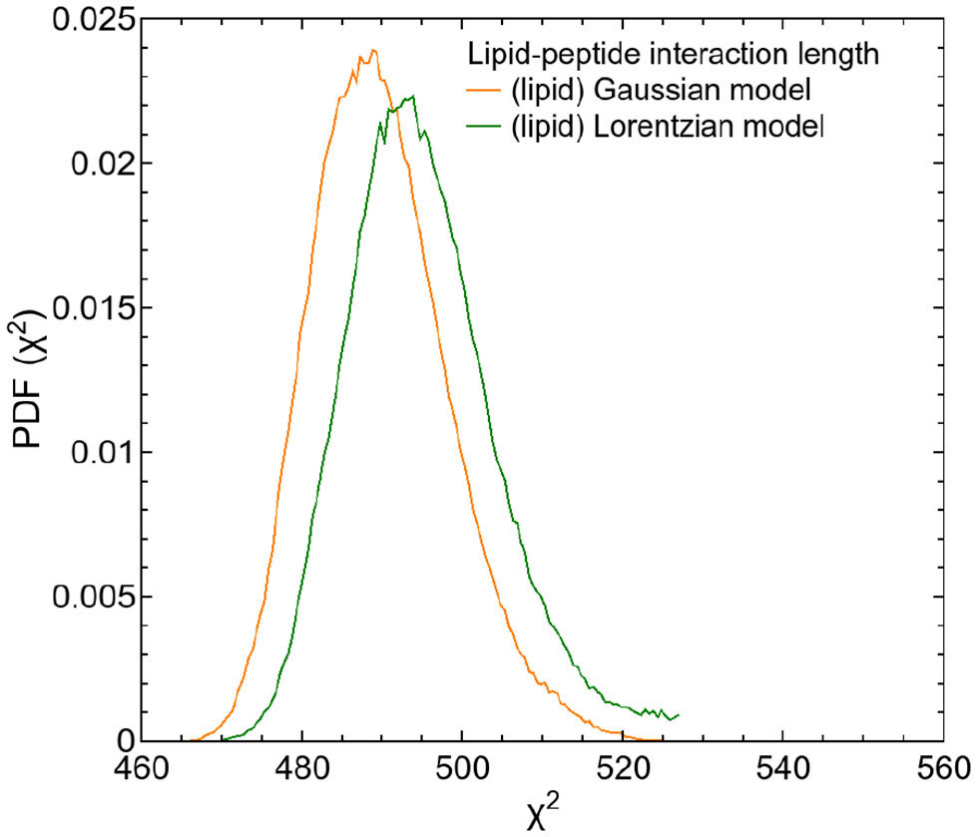
PDFs as a function of χ^2^ for both fit models of the respective DMPC/TFRC data. The PDF of the fit of the Gaussian model is displayed in orange, the one of the Lorentzian model is displayed in green.

The first of these additive terms (second term in Equation 7) describes the proportion *l* of non-interacting lipids. The parameters of this term are adopted from the fits to the QENS data of pure DMPC vesicles. The second term represents the proportion (1 − *l*) of lipids with dynamics slowed down by interaction with the peptide. The parameters describing the peptide dynamics within this term were fixed to the values of the previous results. A fit to the data allows to determine the proportion *l* of the affected lipids and their average velocity *v*_0_ by using σ_lip, 2_ = ℏ*v*_0_*Q*. The diffusion model (subsection 2.3) can be extended analogously by replacing the Gaussians *G*(σ_lipid,1_,ω) and *G*(σ_lipid,2_,ω) in Equation (7) by the corresponding Lorentzians *L*(Γ_lipid,1_,ω) and *L*(Γ_lipid,2_,ω). In order to calculate a lipid-peptide interaction length, both, the Gaussian and the Lorentzian model for the lipid dynamics were tested. In both analysis, the protein dynamics were described by the Lorentzian model. The corresponding PDFs of the fits are displayed as a function of χ^2^ in Figure 9.

**Figure 10:**
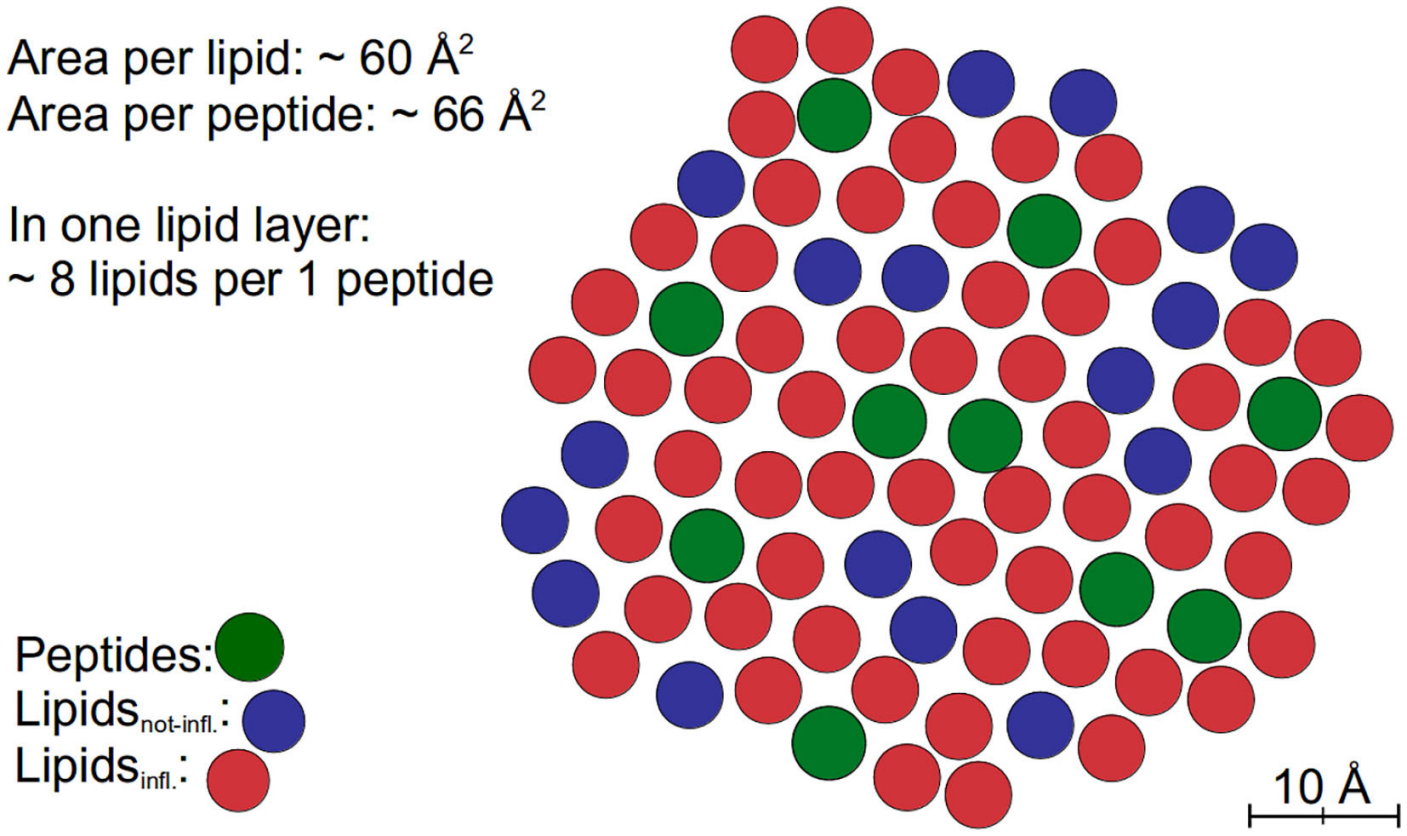
Schematic representation of a fraction of a lipid layer with peptides in top view. The peptides are colored green, the lipids interacting with the peptides (Lipids_infl._) are labeled red, and other lipids (Lipids_not-infl._) are labeled blue.

**Figure 11:**
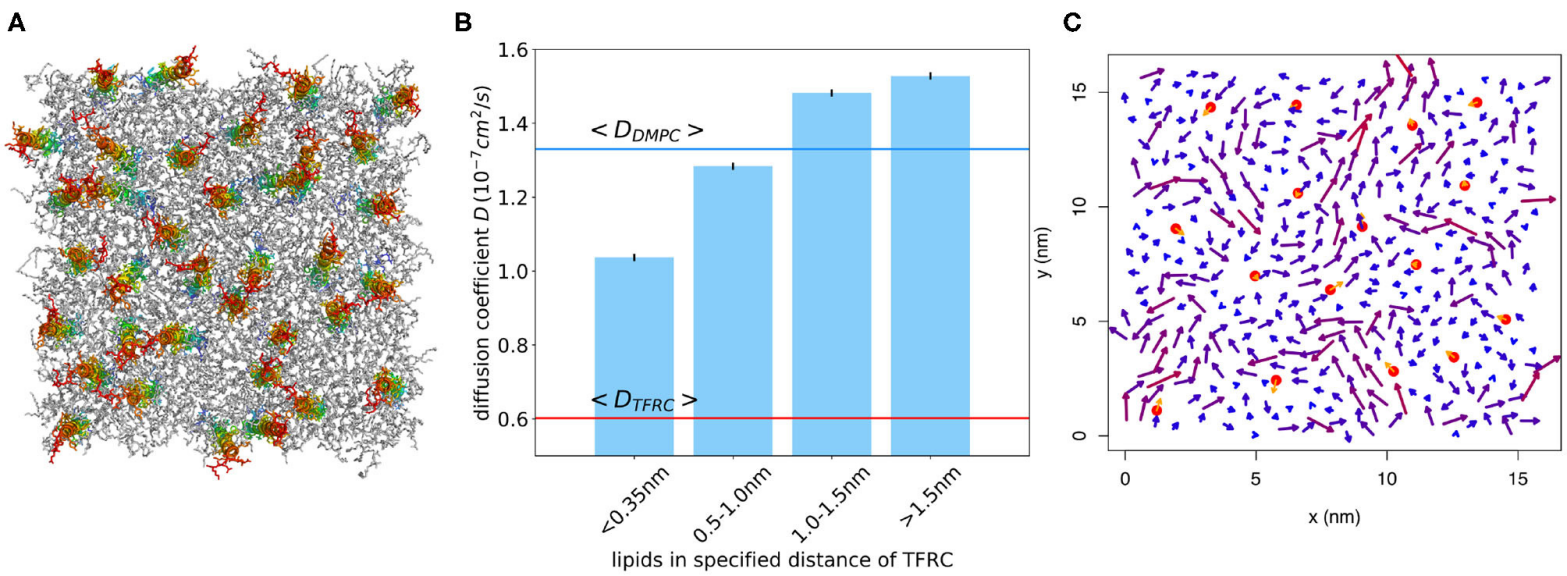
**(A)** Snapshot of atomistic MD simulation of a DMPC/TFRC system at 6 mol% TFRC after 500 ns equilibration (lipids in gray, peptides colored). **(B)** Lipid diffusion coefficient for different distances from the peptide for the system at 2 mol% TFRC. The diffusion coefficient was determined from time windows of 10 ns length. For comparison, the average lipid and TFRC diffusion coefficients are displayed as blue and red horizontal lines, respectively. **(C)** The arrows represent the peptide (orange) and lipid (blueish) average molecular center of mass velocity for a selected time window of 10 ns, respectively. Lipids in the vicinity of a peptide move considerably slower on average.

The extended models can nicely be fitted to the QENS data. The results of both tested models indicate that the dynamics of about 80% of the lipids are influenced by the peptides and exhibit a reduced lateral long-range dynamics. Assuming an effective area *A*_DMPC_ ≈ 60 Å^2^ of a DMPC molecule and an effective area *A*_TFRC_ ≈ 66 Å^2^ of a TFRC peptide in the membrane as calculated from our MD atomistic structures, an average peptide-lipid interaction radius between 1 and 1.5 nm around the peptide can be estimated. Based on this knowledge, the peptide-lipid interaction length has been visualized by a schematic representation of a single lipid layer in top view in Figure 10. In the scheme it becomes obvious that the dynamics of nearest lipid neighbors of a peptide but also some second next neighbors are influenced by the peptide. Beyond that, at the relatively high peptide concentration of 6 mol% most of the lipids are within the peptide-lipid interaction radius of about 1.5 nm (cf. Figure 11A).

In order to further quantify the interaction radius of TFRC, an additional 500 ns atomistic MD simulation of a DMPC/TFRC system has been performed at a reduced TFRC density of 2 mol%. Despite the smaller peptide concentration, the lipid diffusion coefficient was reduced by about 30% with respect to the pure DMPC (cf. Table 2). The mobility of the lipids in close vicinity (phosphate within distance *d* < 0.35 nm) of the peptides was found to be drastically reduced (Figure 11B). For short times (*t* < 10 ns) lipids within the first shells moved together with the transmembrane peptides (Figure 11C). Overall, the simulations confirm an interaction radius of 1–1.5 nm around transmembrane peptides. A similar interaction radius was reported before for the Kv1.2 protein (Niemelä et al., 2010).

## 4. Conclusion

QENS experiments and MD simulations have been performed to study the long-range molecular motions (covering several lipid-lipid distances) within a DMPC membrane loaded with TFRC peptides in transmembrane orientation. The results demonstrate that QENS is capable to separately explore the lipid and peptide long-range motions and to quantify both the peptide lateral self-diffusion coefficient and its influence on the lipid long-range mobility. The calculated self-diffusion coefficients for the lipids are in excellent agreement with the results of state-of-the-art MD simulations and other experimental investigations. The significantly lower diffusion coefficients for TFRC as compared to our simulations further hints to the formation of peptide-rich domains harboring also most of the lipids (~80%) that are characterized by slow dynamics and possibly also increased ordering. Using a consistent fitting model for the QENS data, it was possible to determine the peptide-lipid interaction length within the membrane to be about 1–1.5 nm. This finding is in perfect agreement with results from MD simulations of TFRC molecules embedded in a DMPC bilayer.

## Data Availability Statement

The raw data supporting the conclusions of this article will be made available by the authors, without undue reservation.

## Author Contributions

LE performed the QENS measurements, did the data analysis, and wrote the manuscript. TSc helped during the QENS experiments. SK and KP performed the MD simulations and analysis. AS contributed with help on TFRC selection, preparation, and purification. TSe supported us during the QENS measurements and provided data reduction. RB helped with MD simulation data analysis. TU supported with QENS data interpretation. RB and TU contributed to manuscript writing and organized financial support. All authors did proof reading of the manuscript.

## Conflict of Interest

The authors declare that the research was conducted in the absence of any commercial or financial relationships that could be construed as a potential conflict of interest.
